# Supplementary figures and images for: Mining the Modular Structure of Protein Interaction Networks
Source: PLoS One. 2015 Apr 9;10(4):e0122477. doi: 10.1371/journal.pone.0122477 (PMC4391834; doi:10.1371/journal.pone.0122477)

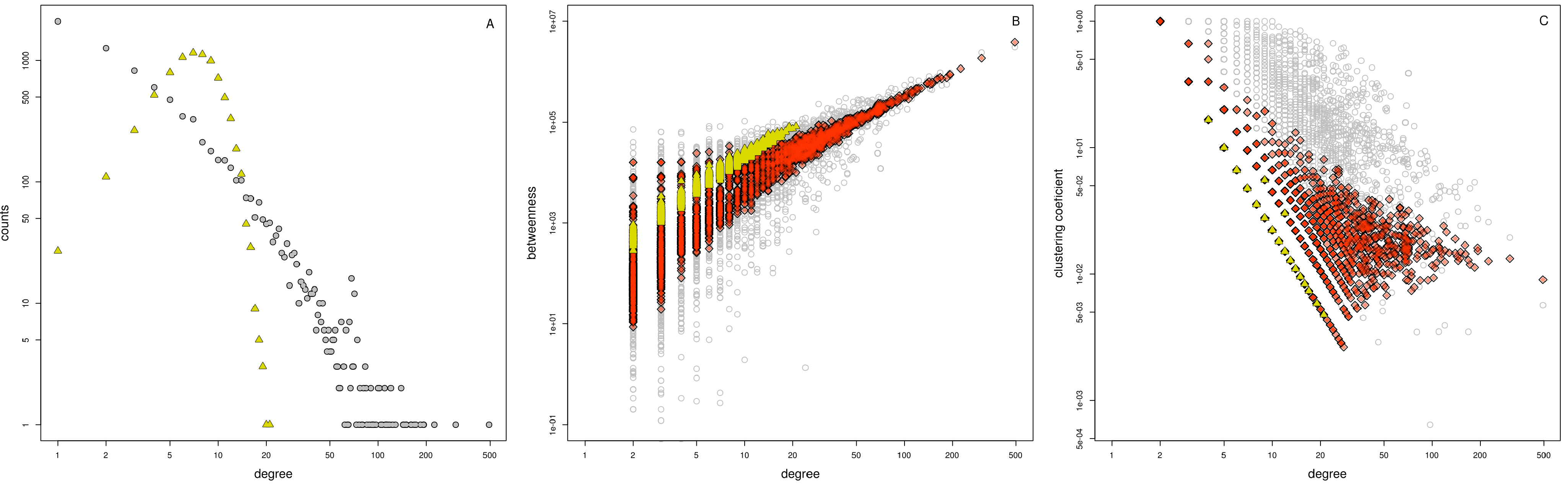

Supplement: S1 Fig — (A) node degree distribution for HIPPIE network (gray circles), and for the respective Erdos-Renyi random network (yelow triangles). Nodes of the HIPPIE network (gray circles), Rewired Network (red diamonds) and Erdos-Renyi network (yellow triangles) were displayed over the Degree-Betweenness and the Degree-Clustering Coefficient planes in panels (B) and (C) respectively. (TIF) [file pone.0122477.s001.tif]

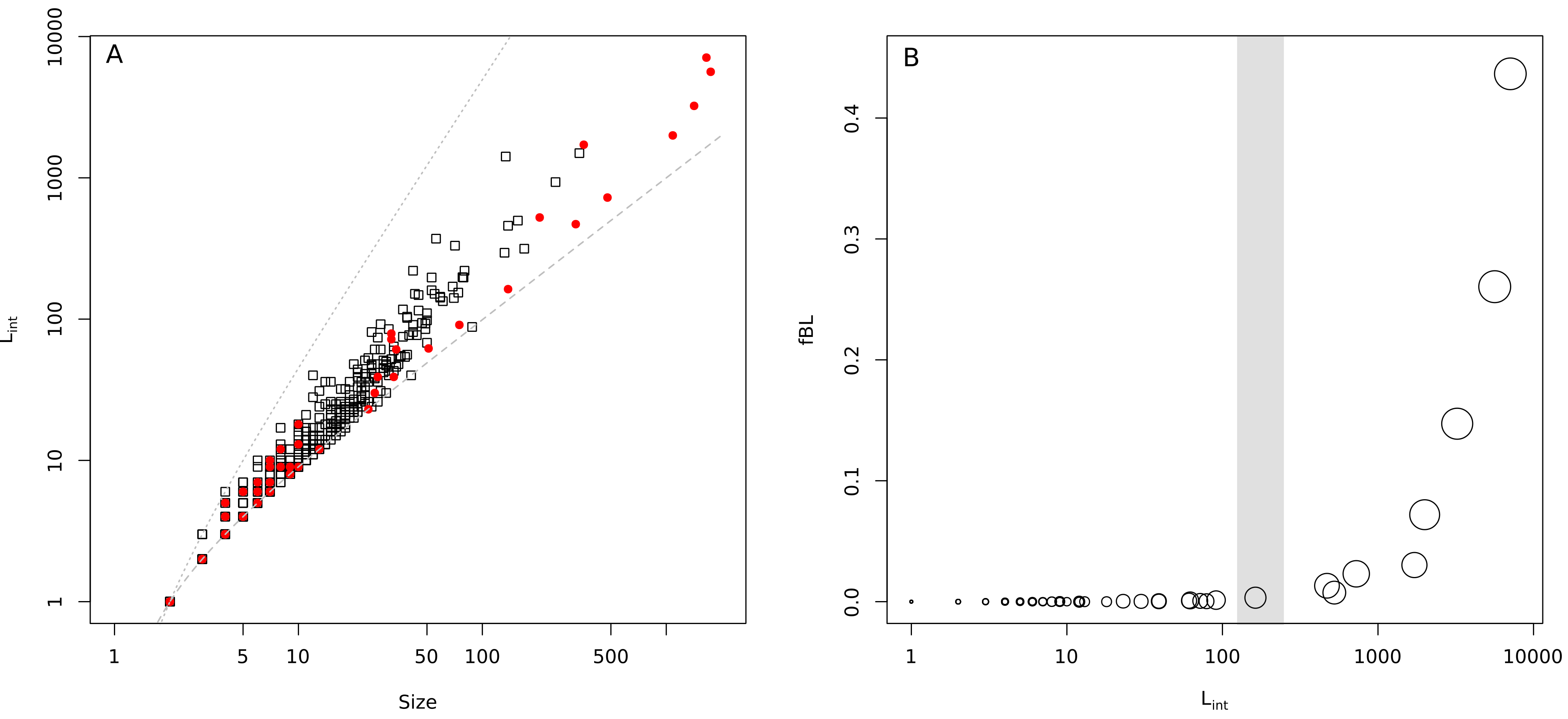

Supplement: S2 Fig — (a) Number of internal links, lint, as a function of cluster sizes. The dotted and dashed lines depict the expected relationships for fully connected cliques and linear structures respectively, and are included for reference purposes. (b) Fraction of internal CNM cluster’s links that did not appear as internal links in the infomap modular description (i.e. fraction of broken links, fBL). Circle sizes are proportional to each CNM cluster log-size. (TIF) [file pone.0122477.s002.tif]

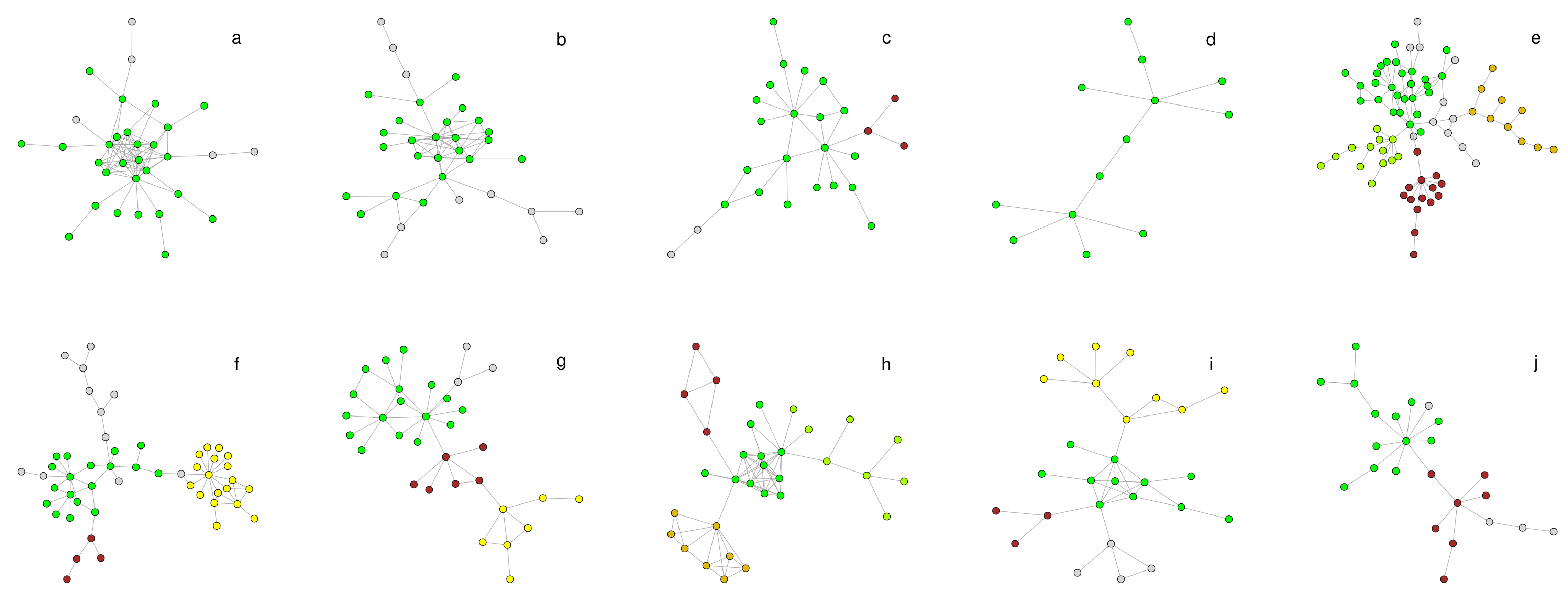

Supplement: S3 Fig — CNM clusters with low internal-link density and more than ten nodes. infomap communities were depicted using different colors. Gray colored nodes belonged to infomap clusters not-totally included in the displayed CNM structure. Two scenarios can be recognized. For cases (a)-(d) a rather good agreement between the alternative modular descriptions was observed. However, for the cases illustrated in panels (e)-(j) internal structure not resolved by the CNM procedure was indeed highlighted by the infomap prescription. (TIF) [file pone.0122477.s003.tif]

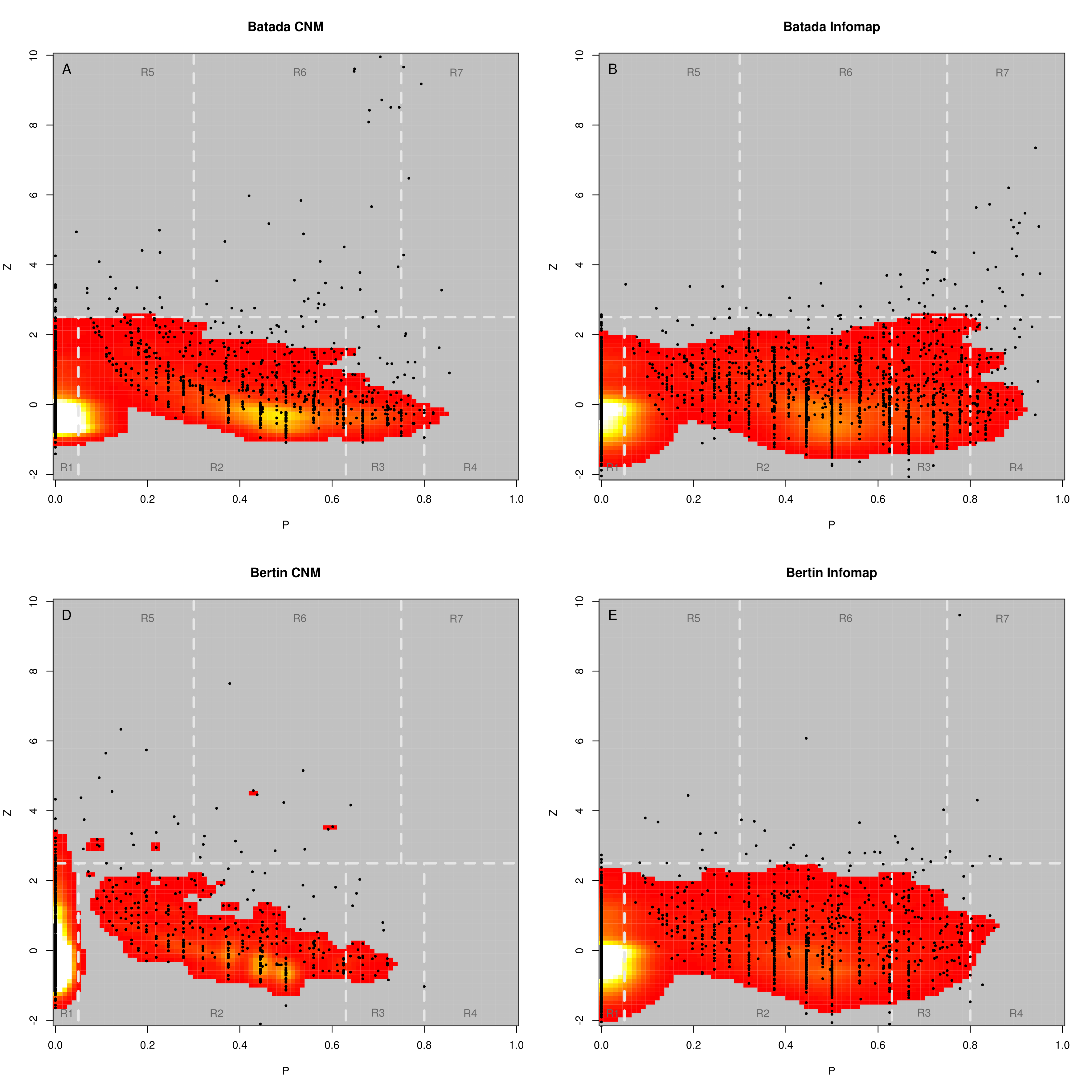

Supplement: S4 Fig — Z-P planes for Batada yeast PPI network (panels A-B) and Bertin yeast PPI Network (panels C-D). Left and right panels correspond to CNM-based and infomap-based cartographical descriptions respectively. An overall increasing behavior in node participation levels can be observed when the infomap cluster recognition procedure was considered. (TIF) [file pone.0122477.s004.tif]

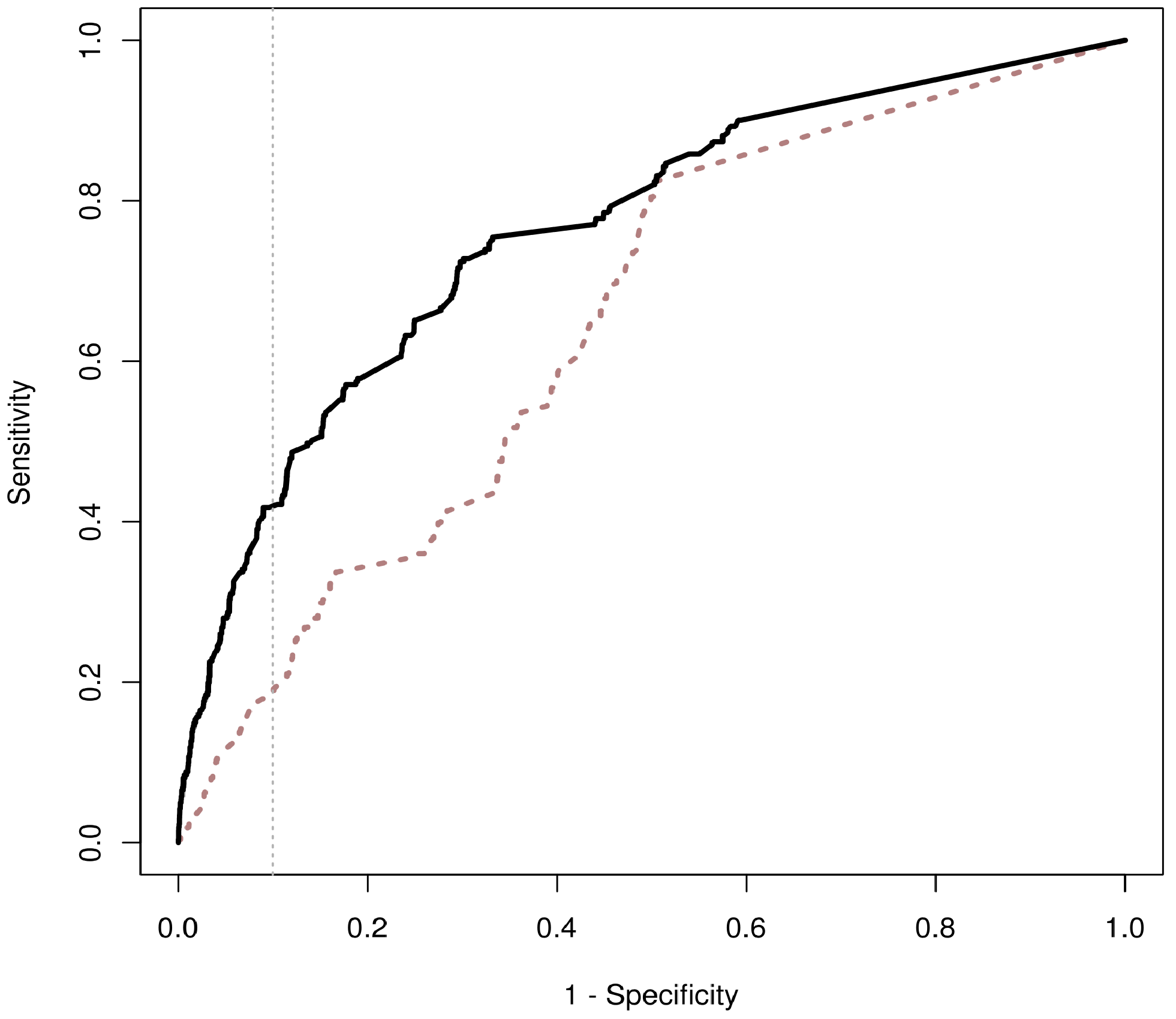

Supplement: S5 Fig — Participation-based ROC curves estimated for ARG genes for CNM and infomap modular descriptions are shown with dashed brown and continuous black lines respectively. The vertical dotted line represents the 90% specificity level. Statistically significant differences between total AUCs are observed (AUC-IFM = 0.76; AUC-CNM = 0.65; pvalue< e-16, deLong’s test), suggesting that the resolution level provided by infomap enhanced the detection of the topological bias displayed by ARG genes toward high-participation levels. (TIF) [file pone.0122477.s005.tif]

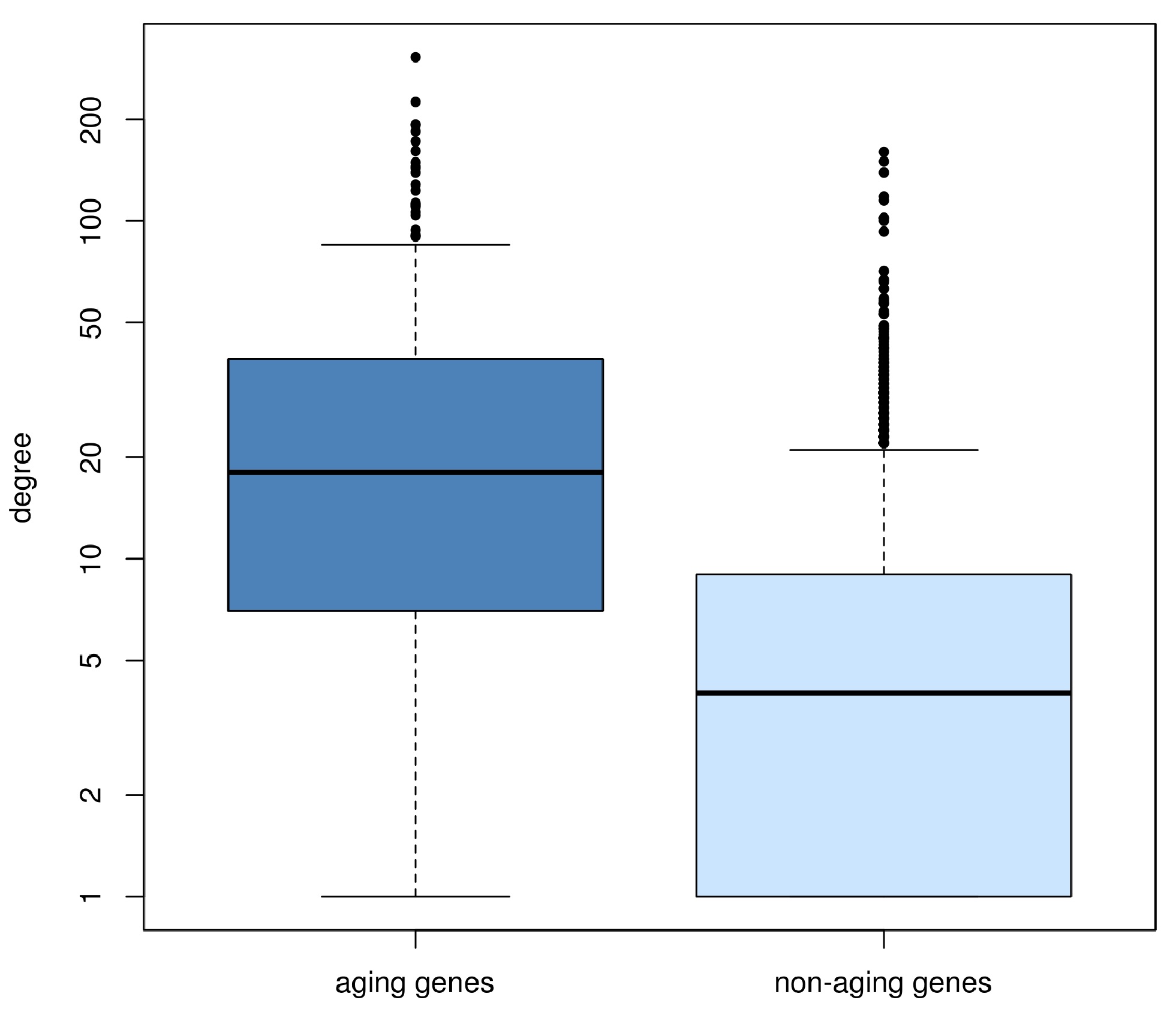

Supplement: S6 Fig — The degree distribution of aging genes, and the whole HIPPIE network remaining nodes are shown in the left and right boxplots respectively. Significant differences (pv< e-16, Wilcoxon test) were observed between both degree distributions. (TIF) [file pone.0122477.s006.tif]

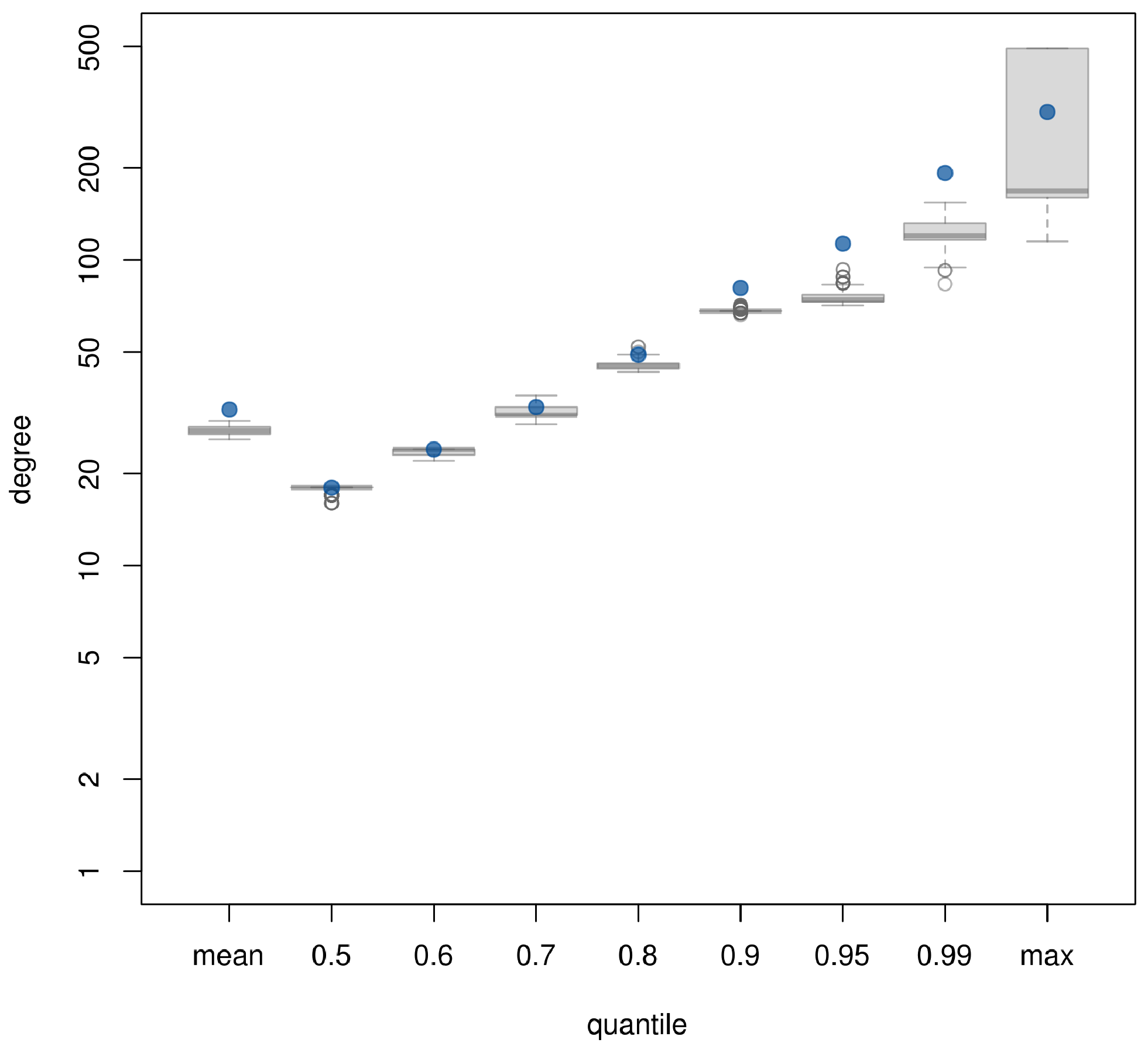

Supplement: S7 Fig — Degree distributions for selected quantiles of 1000 control random realizations are displayed as boxplot. Blue circles depict ARG degree values for the respective quantiles. It can be observed that the top-10% of ARG with highest degree levels, lay outside the inter-quartile levels of their corresponding control random samples. (TIF) [file pone.0122477.s007.tif]

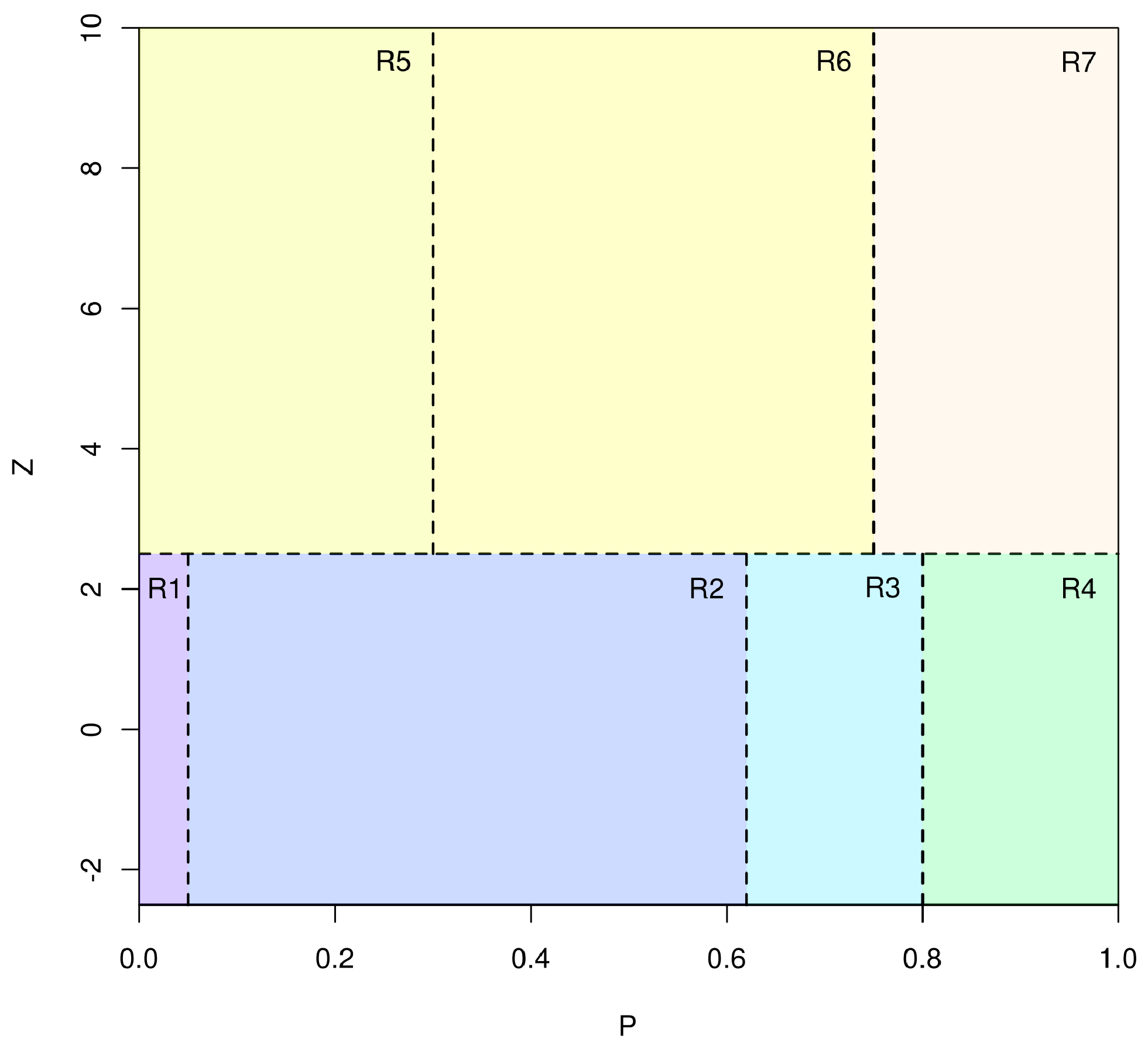

Supplement: S8 Fig — (TIF) [file pone.0122477.s008.tif]
