# Supplementary material for: Mining the Modular Structure of Protein Interaction Networks
Source: PLoS One. 2015 Apr 9;10(4):e0122477. doi: 10.1371/journal.pone.0122477 (PMC4391834; doi:10.1371/journal.pone.0122477)
Supplement: S1 Table — Distribution of cartographic role assignments according to the Infomap and CNM descriptions. Cartographic role abbreviation: Ultra peripheral (R1), Peripheral (R2), Connector (R3), Kinless (R4), Provincial Hubs (R5), Connector Hubs (R6), Kinless Hubs (R7). The coarser resolution level achieved by the CNM algorithm resulted in a general tendency to assign lower participation coefficient values to network nodes. For instance 68% of infomap-connector vertices were assigned to lower participation roles (59% peripheral, 9% ultra-peripheral) when the CNM procedure was considered. More strikingly, the majority (94%) of the 635 infomap-kinless nodes were re-classified as: CNM-connectors (45%), CNM-peripheral (48%), and CNM-ultra-peripheral nodes (1%). Finally, it can also be observed that nodes originally assigned to hub-like roles when infomap procedure was employed were not only affected by this lowering effect in the participation, but in addition, almost 50% of them were also reassigned to non-hub roles when CNM was considered. (DOCX) [file pone.0122477.s009.docx]

**Table S1:** Distribution of cartographic role assignments

|  | ***CNM*** | | | | | | | | |
| --- | --- | --- | --- | --- | --- | --- | --- | --- | --- |
| ***Infomap*** |  | ***R1*** | ***R2*** | ***R3*** | ***R4*** | ***R5*** | ***R6*** | ***R7*** | ***Total*** |
|  | ***R1*** | 3009 | 152 | 1 | 0 | 0 | 0 | 0 | 3162 |
|  | ***R2*** | 716 | 1497 | 84 | 0 | 18 | 32 | 0 | 2347 |
|  | ***R3*** | 146 | 936 | 500 | 0 | 7 | 21 | 0 | 1610 |
|  | ***R4*** | 9 | 304 | 288 | 11 | 4 | 19 | 0 | 635 |
|  | ***R5*** | 3 | 6 | 0 | 0 | 3 | 0 | 0 | 12 |
|  | ***R6*** | 1 | 44 | 5 | 0 | 18 | 34 | 0 | 102 |
|  | ***R7*** | 0 | 25 | 31 | 2 | 8 | 64 | 2 | 132 |
|  | ***Total*** | 3884 | 2964 | 909 | 13 | 58 | 170 | 2 | 8000 |

Distribution of cartographic role assignments according to the Infomap and CNM descriptions. Cartographic role abbreviation: Ultra peripheral (R1), Peripheral (R2), Connector (R3), Kinless (R4), Provincial Hubs (R5), Connector Hubs (R6), Kinless Hubs (R7). The coarser resolution level achieved by the *CNM* algorithm resulted in a general tendency to assign lower participation coefficient values to network nodes. For instance 68% of *infomap*-connector vertices were assigned to lower participation roles (59% peripheral, 9% ultra-peripheral) when the CNM procedure was considered. More strikingly, the majority (94%) of the 635 *infomap*-kinless nodes were re-classified as: CNM-connectors (45%), CNM-peripheral (48%), and CNM-ultra-peripheral nodes (1%). Finally, it can also be observed that nodes originally assigned to hub-like roles when *infomap* procedure was employed were not only affected by this lowering effect in the participation, but in addition, almost 50% of them were also reassigned to non-hub roles when CNM was considered
