# Supplementary material for: Mining the Modular Structure of Protein Interaction Networks
Source: PLoS One. 2015 Apr 9;10(4):e0122477. doi: 10.1371/journal.pone.0122477 (PMC4391834; doi:10.1371/journal.pone.0122477)
Supplement: S1 Text — (DOCX) [file pone.0122477.s010.docx]

# Supplementary Material

**Mining the modular structure of protein interaction networks**

Ariel José Berenstein*^1^, Janet Piñero*^2^, Laura Inés Furlong^2^, Ariel Chernomoretz ^1,3^

**Text A**

#### *Protein interaction network characterization*

We began our analysis summarizing main general topological features related to the *degree* distribution, *clustering coefficient* [1], and *betweenness* [2] of the nodes of the considered HN. These quantities asses for the number of neighbors, the connectivity among them, and the node relevance in terms of global information flux over the entire network, respectively.

A network characterization in terms of node degree distribution involved one of the most basic and intuitive connectivity-related notion of centrality. The protein interaction network exhibited a heavy-tailed empirical degree distribution - gray points in Figure S1 - reflecting large fluctuations in vertex connectivity (while the network nodes presented an average degree of $\acute{k}=7.7$, the maximum degree was *k_max_=492*).This feature is not recapitulated in the ERN null model (see the Method section for a brief description of the considered network null models) - yellow points in Figure S1a - for which the existence of a natural scale for vertex connectivity can be recognized. This fundamental discrepancy has already been widely reported for many different real world networks, and highlights the existence of non-trivial correlation patterns at the level of vertices connectivity.

The heterogeneity observed in neighbor numbers implies the existence of unusually highly connected hub nodes that could act as general shortcuts, globally shortening geodesic distances over the entire network. However, in terms of global information spreading capabilities, additional non-trivial relevant network nodes might also exist. The *betweenness* centrality concept aims to exploit this information-flux point of view to characterize structural properties of network nodes. Figure S1b displays the node’s *betweenness* as a function of the node’s degree for HN, ERN, and RWN (shown as gray, yellow and red circles respectively). A monotonic and increasing relationship can be recognized for all the three considered networks, denoting the somehow expected general positive correlation trend that exists between the degree and betweenness of a node. Noticeably, for a given degree value, the bio-molecular network presented a wider distribution of betweenness levels. In addition, it presented a higher fraction of high-betweenness and low-degree nodes than the two alternative random network models. If only low connectivity vertices (e.g. having less $\acute{k}=7.7$ neighbors) were considered, forty nine nodes would be found within the top-10% betweenness score ranking in HN, while four and none in the RWN and ERN cases respectively. These *bottleneck* nodes, that are overrepresented in the real network, constitute an *a priori* interesting subset of proteins since they could play a central intermediary role in information transmission processes taking place over the entire network [2,3] .

Insights about local connectivity patterns can also be gained looking at the node’s *clustering coefficient* that quantifies the connectivity among first neighbors of a given node. Figure S1cdisplays the *clustering coefficient* as a function of the node’s degree for HN, ERN, and RWN (shown as gray, yellow and red circles respectively). An expected general negative correlation trend can be observed between these two quantities for the three networks. However a much larger number of nodes presenting high clustering coefficient values can be observed for HN.

The presented results suggested the existence of non-trivial topological heterogeneities compatible with a putative underlying modular organization in HN, our bio-molecular network of interest, similarly to what have already been reported in other biological inspired network-based analysis [4-6]. Moreover, the observed structural differences with respect to randomized networks allow us to anticipate that HN interconnectivity patterns probed by different topological observables could highlight non-trivial network components, such us high-betweenness, low-connectivity proteins that might act as important links between modular structures.

#### *Text B*

#### *Clustering procedures*

The *CNM* algorithm [7] looks for communities by direct optimization of the modularity *Q* of the graph, that is defined, up to a multiplicative constant, as the number of edges falling within groups minus the expected number in an equivalent control network with edges placed at random. As stated in the main text, when the considered null network model preserves the degree distribution of the original network, Q can be expressed as:

$Q=\frac{1}{2L}\sum_{ij}^{N} \left( A_{ij}-\frac{k_{i}k_{j}}{2L} \right)\delta\left( C_{i},C_{j} \right)$ [1]

In the above equation, $k_{i}$is the degree of node-*i*, *L* is the total number of network edges, *N* the total number of nodes, $A_{ij}$ is the adjacency matrix of the network (*A_ij_=1* if there is a link between nodes *i* and *j*, and zero otherwise). *C_i_* identifies the community that includes node-*i*, and $\delta\left( C_{i},C_{j} \right)$ is a delta function (i.e. $\delta\left( C_{i},C_{j} \right)=1$ if node-i and node-j belong to the same cluster, and zero otherwise). Note that the expected number of edges between vertices *i* and *j* if edges are placed at random is $k_{i}k_{j}/2L.$

On the other hand, the *infomap* algorithm relies on very different optimization criteria. Clusters are defined in order to minimize the average description length of random walk process taking place over the graph. A two level hierarchy, involving a community tag and a within-community ID tag,is used to identify each network node. As random walkers are expected to expend a lot of time inside densely structures in the graph (i.e. communities), the algorithm iteratively search for node-tagging schemes that produce increasingly compact descriptions of the random walk process. As a by product,a sensible description of the network modular structure is achieved. The *infomap* objective function can be thought in terms of the entropy associated to the random walk process and involved two contribution terms. The first one represents the entropy of the movement between modules, while the second one corresponds to movements within modules:

$L\left( P \right)=q_{inter}H\left( Q \right)+\sum_{i}^{s} p_{intra}^{i}H\left( P^{i} \right)$ [2]

Here, $q_{inter}$ is the probability that the walker switches clusters,$H\left( Q \right)$is the entropy associated to between clusters transitions,$p_{intra}^{i}$ is the fraction of movements occurring inside cluster *i*, and $H\left( P^{i} \right)$is the entropy of movements within the cluster*i* .[8].

As can be seen from equations (1) and (2) both considered algorithms rely on very different assumptions and optimization criteria, and thus could provide in principle alternative and complementary descriptions of the modular structure of the analyzed network. A more detailed and general description of optimization criteria and performance comparison of both algorithms can be found in [9,10].

#### *Text C*

#### *Biological Homogeneity Index*

Following Datta&Datta[11], we considered a partition of *k* clusters,$\{D_{1},D_{2},\ldots,D_{k}\}$, and assumed that *C(x)* is a functional class containing gene *x*. The biological homogeneity index of the partition resulted:

$$BHI=\frac{1}{k}\sum_{j=1}^{k} \frac{1}{n_{j}(n_{j}-1)}\sum_{x\neq y\in\mathcal{D}_{j}} I(C\left( x \right)=C\left( y \right))$$

where*n_j_* is the size of cluster-*j.* The indicator function *I(C(x) = C(y))* takes the value 1 if *C(x)* and *C(y)* match. We made use of the functionality implemented in the *clValid* R-package[12], and disregarded functional GO classes annotated under the IEA (inferred from electronic annotations) evidence code.

#### *Text D*

#### *Degree-aware bootstrap for topographic role enrichment*

A bootstrapping procedure was devised for the topographic role enrichment analysis of the considered gene-groups in order to control for the node’s degree distribution factor. For each enrichment test, we considered an ensemble of 1000 control random gene-sets having the same degree distribution than genes under study, and a p-value level was estimated according to the number of random realizations displaying the same or larger effects (over/under representation significance) than the ones observed in the original data.

Each random realization was conformed according the degree displayed by the original gene set randomly extracting genes from pools of given degree levels. In order to warrant for a non-biased sampling, we binned by degree the available sampling nodes requiring a minimal sample size of 100 nodes per bin. Once the non-uniform binning was established we made an *a-posteriori* analysis to make sure that the degree distributions of control random realizations had similar statistical features than the observed one. To that end, we identified which quantile level of the original data was not duly sampled looking for cases where the corresponding observed degree were not included in the inter-quartile range of the respective control realizations. For instance, it can be appreciated form figure S7 that the high degree level of the top 10% of the ARG set, could not be reproduced by the random sampling procedure. In this case the bootstrap analysis was performed considering a reduced ARG’ set, discarding the 10% most connected aging related genes.

Bibliography

1. Watts DJ, Strogatz SH. Collective dynamics of “small-world” networks. Nature. 1998;393:440–2.

2. Freeman LC. A Set of Measures of Centrality Based on Betweenness. Sociometry [Internet]. 1977;40:35. Available from: http://www.jstor.org/stable/3033543?origin=crossref

3. Yu H, Kim PM, Sprecher E, Trifonov V, Gerstein M. The importance of bottlenecks in protein networks: Correlation with gene essentiality and expression dynamics. PLoS Comput Biol. 2007;3:713–20.

4. Barabási A-L, Oltvai ZN. Network biology: understanding the cell’s functional organization. Nat Rev Genet. 2004;5:101–13.

5. Guimerà R, Sales-Pardo M, Amaral LAN. Module identification in bipartite and directed networks. Phys Rev E - Stat Nonlinear, Soft Matter Phys. 2007;76.

6. Cai JJ, Borenstein E, Petrov DA. Broker genes in human disease. Genome Biol Evol. 2010;2:815–25.

7. Clauset A, Newman M, Moore C. Finding community structure in very large networks. Physical Review E. 2004.

8. Rosvall M, Bergstrom CT. Maps of random walks on complex networks reveal community structure. Proc Natl Acad Sci U S A. 2008;105:1118–23.

9. Lancichinetti A, Fortunato S. Community detection algorithms: A comparative analysis. Physical Review E. 2009.

10. Lancichinetti A, Fortunato S. Limits of modularity maximization in community detection. Phys Rev E - Stat Nonlinear, Soft Matter Phys. 2011;84

11. Datta S, Datta S. Methods for evaluating clustering algorithms for gene expression data using a reference set of functional classes. BMC Bioinformatics. 2006;7:397.

12. Brock G, Pihur V, Datta S, Datta S. clValid : An R Package for Cluster Validation. J Stat Softw [Internet]. 2008;25:1–28. Available from: http://citeseerx.ist.psu.edu/viewdoc/download?doi=10.1.1.101.3573&amp;rep=rep1&amp;type=pdf
